# Screening factors affecting proper levothyroxine therapy among patients with primary hypothyroidism: a cross-sectional study

**DOI:** 10.25122/jml-2023-0387

**Published:** 2024-02

**Authors:** Zainab Hussein Ali, Manal Khalid Abdulridha, Qusay Baqer Alzajaji

**Affiliations:** 1 Department of Clinical Pharmacy, College of Pharmacy, Kerbala University, Kerbala, Iraq; 2 Department of Clinical Pharmacy, College of Pharmacy, Mustansiriyah University, Baghdad, Iraq; 3 Alhassan Metabolism, Endocrine and Diabetes Center (HMEDC), Karbala, Iraq

**Keywords:** primary hypothyroidism, KAP, adherence, depression, TSH

## Abstract

Primary hypothyroidism, the most common form of hypothyroidism, requires effective patient understanding and management for successful long-term treatment. This study aimed to investigate the influence of patient knowledge, attitude, practice (KAP), depression, and medication adherence on treatment response in primary hypothyroidism. A cross-sectional observational study was conducted at Al Hassan Metabolism, Endocrine, and Diabetes Center (HMEDC) in Iraq between September 2022 and March 2023. We enrolled 111 patients with signs and symptoms of primary hypothyroidism over 6 months. A validated questionnaire assessed patient knowledge, attitude, practice (KAP), depression, and medication adherence. Thyroid-stimulating hormone (TSH) levels were measured to assess treatment response. Data were analyzed using SPSS v26, with categorical variables presented as percentages. The student's t-test was used to assess statistical significance, with *P* - valuess below 0.05 considered significant and *P* - values below 0.01 considered highly significant. The mean age of patients was 45 ± 11.9 years. Approximately 34% of patients had insufficient knowledge, and 30% indicated a positive attitude towards their treatment. A total of 35% of patients had excellent practice. There was no statistically significant association between KAP and age or gender. There was a significant positive correlation between higher levels of education and improved KAP scores. A total of 44.1% of participants reported moderate depression, and 58% demonstrated adherence to levothyroxine (LT4) treatment. Despite good adherence, the combination of fair knowledge and moderate-to-severe depression resulted in suboptimal outcomes for replacement treatment.

## INTRODUCTION

The thyroid gland plays a crucial role in maintaining homeostasis, promoting growth, facilitating brain development, and supporting accurate functioning in the neurological and circulatory systems [1]. Hypothyroidism is a prevalent endocrine illness with a global impact, necessitating treatment in extreme instances. Inadequate management of hypothyroidism has been associated with adverse health outcomes, including infertility, cardiovascular illness, as well as neurological and musculoskeletal issues [2]. The clinical manifestations associated with hypothyroidism include symptoms such as increased body weight, persistent tiredness, feeling cold in the extremities, depression or anxiety, bowel problems, headaches, irregular menstrual cycles, cognitive difficulties, and dry skin and nails [3]. For patients with chronic conditions like hypothyroidism, a deep understanding of their disease and treatment options is crucial for long-term success and adherence to medication regimens. Hypothyroidism presents various symptoms that healthcare professionals should be mindful of during consultations, such as weight gain, fatigue, and depression [3,4]. A recent study suggests a link between hypothyroidism and depression, possibly due to the involvement of hormones like somatostatin and serotonin in the hypothalamus-pituitary-thyroid axis [5]. Polypharmacy and a significant degree of co-morbidity, especially cognitive co-morbidity, make adherence even more difficult. Patients, especially those with cognitive comorbidities, may find the complex regimen of levothyroxine (LT4), which sometimes requires dosage adjustments on different days of the week for proper titration, particularly challenging to follow. Polypharmacy also makes it harder to achieve optimal absorption. For example, people with dementia may have trouble taking levothyroxine separately from other prescriptions and on an empty stomach, as normally suggested. Strategies such as using pill organizers or scheduling weekly doses have been proposed to mitigate these issues, yet the effectiveness of these approaches, including the potential benefits of a bedtime dose separate from other medications, remains uncertain [6]. The administration of a daily dosage of LT4 is sufficient for the efficient management of hypothyroidism. However, several factors may influence the absorption of levothyroxine, including age, weight, other medical conditions, and dietary intake [7].

The understanding and management of illnesses are significantly influenced by an individual's knowledge, attitudes, and practices (KAP). Uncertainty about the risks of skipping medication can make it harder for both healthcare providers and patients to manage the condition. Many patients are unaware of the potential genetic link to hypothyroidism, which can leave them uninformed about this aspect of their disease [8]. Counseling services play a crucial role in enhancing patients' understanding of hypothyroidism and the administration of thyroxine replacement medication, thereby contributing to the optimization of pharmacotherapeutic results. This optimization can be measured by examining three key factors: knowledge, attitude, and practice (9). Providing patients with comprehensive information on their illness, equipping them with effective coping strategies, and guiding them toward adopting appropriate lifestyle modifications are crucial elements in facilitating symptom management, reducing the likelihood of complications, enhancing treatment results, and reducing morbidity rates [10]. Patient education, including assessments of knowledge and counseling sessions, plays a crucial role in improving treatment outcomes for hypothyroidism [9, 11]. Effective communication can help reduce medication-related issues and enhance patients' physical, social, and emotional well-being by providing targeted medical advice on managing the condition [12]. Existing research acknowledges a significant gap in the overall knowledge and awareness regarding hypothyroidism. This lack of awareness is often associated with inadequate comprehension, misunderstandings, and suboptimal practices among a considerable number of individuals experiencing this disease [13].

The primary objective of this study was to investigate how various factors, including patient’s knowledge, attitude, practice (KAP), depression, and adherence, influence the effectiveness of hypothyroidism therapy. This research is the first nationwide investigation into the parameters influencing the appropriate administration of levothyroxine replacement therapy, specifically concerning patient-related characteristics.

## MATERIAL AND METHODS

### Study design

This cross-sectional observational study was conducted at Imam Al-Hassan Endocrine Center in Karbala between September 2022 and March 2023 and enrolled patients with signs and symptoms of primary hypothyroidism. The Al Hassan Metabolism, Endocrine, and Diabetes Center (HMEDC) was selected as the research site due to its exclusive availability within the city.

### Sample size

The G*Power software version 3.1.9.7, with the Research Resource Identifier (RRID) SCR_013726, was utilized to estimate the necessary sample size for the study. The study employed a one-tailed alpha level of 0.05, a confidence interval of 95%, a power of 95%, and an effect size of 0.30. Subsequently, the sample size required was determined at 111 (f). Accordingly, the study enrolled 111 individuals.

### Patient selection

The recruitment of patients was entirely voluntary. Participants provided their consent after receiving a comprehensive explanation of the study objectives and assurances of confidentiality, anonymity, and the exclusive use of their data for research purposes. The authors were engaged in a collaborative effort to recruit the participants collectively. A group of 217 individuals was included in the present research. From this group, 106 were excluded due to either a recent diagnosis or non-adherence to treatment protocols, leaving 111 participants for the study.

### Inclusion criteria

- Patients diagnosed with primary hypothyroidism following the relevant clinical guidelines [14].
- Patients aged 18 years or older.
- Patients who received levothyroxine medication for more than three months.

### Exclusion criteria

- Patients recently diagnosed with primary hypothyroidism or other forms of thyroid diseases.
- Patients having surgical procedures, receiving radiation therapy to the neck, or undergoing radioiodine ablation may need other treatments besides levothyroxine.
- Patients with severe mental disabilities or other psychiatric conditions that could impede their ability to complete the study questionnaire.

### Data collection tools

#### Interview

The researchers conducted face-to-face interviews with patients to collect information regarding demographic variables, such as age, gender, body mass index (BMI), education level, family history, and self-reported medical conditions. Information related to the current levothyroxine (LT4) dose and clinical characteristics of individuals were also collected.

### Structured questionnaire

We used four questionnaires to evaluate the knowledge, attitude, and practice (KAP) regarding hypothyroidism [4,15]. In addition, we adapted a questionnaire consisting of nine items from the Patient Health Questionnaire-9, aimed to evaluate the level of depression experienced by patients [16].

### Scoring system

**Knowledge:** This section included 16 items related to the symptoms, risk factors, diagnosis, and management of hypothyroidism. Responses were categorized as either ‘true’, ‘false’, or ‘don't know’. Correct responses were scored one point, while incorrect or unanswered questions were not scored.

**Attitude:** This section had 10 statements about the reasons, dietary restrictions, issues, levothyroxine therapy, medication adherence, and herbal medicine. These statements were used to assess the attitudes of patients towards hypothyroidism and treatment. Correct responses were scored one point, while incorrect or uncertain responses were not scored.

**Practice:** This section included eight practice statements where patients indicated their compliance with a ‘yes’ or ‘no’ response.

To quantify knowledge, attitude, and practice levels, we calculated the percentage of correct answers by dividing the number of correct responses by the total questions and multiplying by 100. Scoring thresholds follow modified Bloom's categorization [17]: above 75% indicates good understanding, 50-75% is fair, and below 50% represents poor knowledge, attitude, or practice. Depression scores were categorized as follows: 5 to 9 indicated mild depression, 10 to 14 moderate, 15 to 19 moderately severe, and 20 to 27 severe. To assess the impact of depression on daily life, patients were asked how it affected their work, home management, and social interactions. Responses were scored as 1 for 'Not difficult at all', 2 for 'Somewhat difficult', 3 for 'Very difficult', and 4 for 'Extremely difficult' [16].

Patient adherence: To evaluate patient adherence to their LT4 treatment regimen, we investigated the frequency at which doses were missed during the previous month. Adherence levels were determined based on the percentage of missed doses and categorized as follows [4,18]:

- Good adherence: Patients who missed less than 5% of their doses in the past month.
- Fair treatment adherence: Patients who missed between 5% and 14% of doses in the last month.
- Poor adherence to treatment: Patients who missed equal to or more than 15% of doses in the previous 30 days.

### Laboratory investigations

#### Thyroid-stimulating hormone (TSH)

The current study used an immunoassay technique to quantitatively measure thyroid-stimulating hormone (TSH) levels in an in vitro setting. The electrochemiluminescence immunoassay, referred to as ECLIA, involves the formation of a sandwich complex comprising two distinct monoclonal antibodies specifically designed to target thyroid stimulating hormone. The device is specifically engineered for compatibility with the Cobas E immunoassay analyzer. The electrode surface has a magnetic attraction towards the microparticles. The chemiluminescent emission resulting from the application of a voltage to the electrode is detected by a photomultiplier. The established range for normal values is 0.5 to 4.5 mIU/L (or µIU/mL) [19].

### Body mass index

The body mass index (BMI) of patients was calculated by dividing their weight in kilograms by the square of their height in meters (kg/m^2^) [20]. Normal weight was defined as a BMI between 18.5 and 25. Overweight was categorized by a BMI of 25 kg/m^2^ or higher, and obesity by a BMI of 30 kg/m^2^ or higher [21]. We further classified obesity based on severity: Obese Class I (BMI 30 to 34.9 kg/m^2^) and Obese Class II (BMI 35 to 39.9 kg/m^2^) [21].

### Statistical analysis

The data collected were initially entered and organized in Microsoft Excel (2016). After cleaning and validation, the data were imported into SPSS software (version 26) for statistical analysis. Numerical data are presented as mean ± standard deviation (SD), and categorical data are presented as percentages. To assess the significance of differences between groups, the Student's t-test was used. A *P* value less than 0.05 was considered statistically significant, and a *P* value less than 0.01 was considered highly significant.

## RESULTS

A total of 111 participants completed the questionnaire. The average age of participants was 45 ± 11.9 years, with 49.5% falling within the age range of 40–60 years (Table 1). Ninety-one percent of the participants included in the study were women. A significant proportion of individuals (36%) had obesity, and 32.4% were classified as overweight. Most patients (45.9%) had a secondary level of education. Treatment initiation varied, around 34% of patients started therapy with a dosage of 50 ug or less, 48% started with a dosage between 75 and 100 ug, and 18% started with a dosage above 100 ug. Past medical records revealed that 65.8% of the participants had a diagnosis of hypothyroidism. The prevalent comorbidities observed were diabetes mellitus (18.9%) and hypertension (27.0%). A significant proportion of patients (68.5%) had slowed speech. Similarly, 64.9% of patients reported constipation, while 18% had a reduced appetite. The majority of patients, almost 73%, demonstrated intolerance to cold, and a substantial 79.3% reported weight increase. Additionally, a substantial percentage of patients (90.1%) experienced fatigue.

**Table 1:** Characteristics of study participants

Variables		*n*	%
**Age(year)**	Mean ± SD	45 ± 11.9	
	< 40 years	42	37.8
	40- 60 years	55	49.5
	> 60 years	14	12.6
**Gender**	Male	10	9.0
	Female	101	91.0
**BMI**	Normal weight	11	9.9
	Overweight	36	32.4
	Obese I	40	36.0
	Obese II	24	21.6
**Education**	Primary	29	26.1
	Secondary	51	45.9
	University	31	27.9
**Dose of levothyroxine**	≤ 50 ug	38	34.0
	75-100 ug	53	48.0
	>100 ug	20	18.0
**Medical history**	Surgical History	74	66.7
	Hypertension	30	27.0
	Diabetes	21	18.9
	Allergy	9	8.1
	Heart Disease	2	1.8
	Drug Interaction	2	1.8
	Immune Disease	1	0.9
**Family history**	Yes	73	65.8
	No	38	34.2
**Signs and symptoms**	Slow Speech	76	68.5
	Constipation	72	64.9
	Loss Appetite	20	18.0
	Cold Intolerance	82	73.9
	Increase weight	88	79.3
	Fatigue	100	90.1

Data presented as number (*n*) and percentage (%)

Table 2 presents the distribution of patients in the study, categorized according to their responses to each knowledge question. A significant proportion of patients (77%) had a good understanding of the anatomy of the thyroid gland. Approximately 89% of individuals were knowledgeable about the definition of hypothyroidism. Most respondents (94%) agreed that levothyroxine should be taken continuously. Moreover, 97% were aware of the importance of consulting an endocrinologist for managing hypothyroidism. Approximately 77% were not aware of the contraindication of concurrent use of thyroid medicines with supplements such as iron and calcium.

**Table 2:** Patient knowledge responses

Questions	True		False		Don’t know	
	*n*	%	*n*	%	*n*	%
The thyroid gland, found in the neck, has the shape of a butterfly.	86	77	8	7	17	15
Hypothyroidism is a medical condition due to low thyroid hormone levels.	99	89	0	0	12	11
Patients with neck swelling or other abnormalities may have hypothyroidism.	47	42	12	11	51	46
Cold intolerance could result from hypothyroidism.	45	41	52	47	14	13
Inherited thyroid problems are not common.	52	47	3	3	56	50
Fatigue is a possible symptom of hypothyroidism.	88	79	0	0	23	21
Constipation is a common symptom of hypothyroidism.	73	66	2	2	36	32
Weight gain could result from hypothyroidism.	88	79	1	1	22	20
Hypothyroidism is diagnosed by measuring TSH, FT4, FT3 levels in the blood	104	94	0	0	7	6
Hypothyroidism has been linked to an inadequate intake of iodine.	26	23	8	7	77	69
People with hypothyroidism may be more likely to experience depression.	33	30	1	1	77	69
Thyroxine pills must be the same brand.	42	38	31	28	38	34
Thyroid medication should be taken indefinitely.	104	94	4	4	3	3
Hypothyroidism requires the care of an endocrinologist.	108	97	0	0	3	3
Thyroid medications shouldn’t be taken at the same time as medications like iron and calcium supplements.	15	14	11	10	85	77
Patients with hypothyroidism may be more likely to experience elevated cholesterol levels.	32	28.8	7	6.3	72	64.9

Data presented as number (n) and percentage (%)

**Figure 1:**
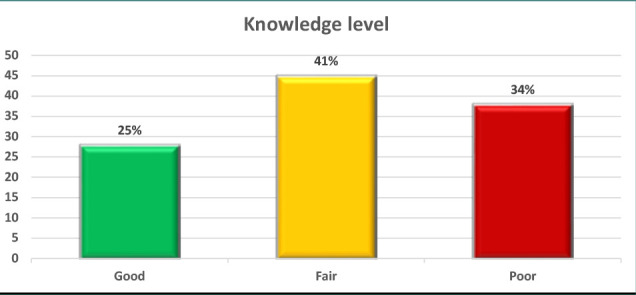
Distribution of participants according to knowledge level

According to the findings in Figure 1, a quarter (25%) of the cases had a high level of knowledge, while a larger proportion (41%) exhibited a moderate level of knowledge. The remaining cases (34%) had a low level of knowledge.

Table 3 illustrates the results regarding patient attitudes towards hypothyroidism treatment and management. Most patients (99.1%) agree that medication adherence is crucial for mitigating symptoms and normalizing thyroid function tests. Moreover, 98.2% agree that therapy should only start after a thorough consultation with a healthcare practitioner regarding available treatment alternatives. Most respondents (95.5%) considered discontinuing thyroxine supplements not advisable. Additionally, 82% of patients had a neutral attitude on the recommendation for individuals aged 35 and older to undergo routine hypothyroidism screenings.

**Table 3:** Patient’s attitude responses

Questions	Agree		Neither agree nor disagree		Disagree	
	*n*	%	*n*	%	*n*	%
Women should have routine thyroid testing since they are more likely to develop hypothyroidism.	84	75.7	25	22.5	2	1.8
People over the age of 35 should be tested frequently for hypothyroidism.	17	15.3	91	82.0	3	2.7
Hypothyroidism screening is recommended for pregnant women.	35	31.5	75	67.6	1	0.9
People should be examined for hypothyroidism if they have relatives or family members who have the condition.	62	55.9	47	42.3	2	1.8
Only after discussing treatment options with a medical professional may hypothyroidism be treated.	109	98.2	2	1.8	0	0.0
If pregnancy is detected, dose titration is required.	26	23.4	72	64.9	13	11.7
Treatment of hypothyroidism with natural remedies is ineffective.	93	83.8	13	11.7	5	4.5
Once the thyroid laboratory profile becomes normal, taking thyroxine supplements should not be stopped.	106	95.5	1	0.9	4	3.6
In order to reduce symptoms and normalize the thyroid test profile, medication compliance is essential.	110	99.1	1	0.9	0	0.0
Self-adjustment of doses is not recommended in hypothyroidism.	104	93.7	3	2.7	4	3.6

Data presented as number (n) and percentage (%)

**Figure 2:**
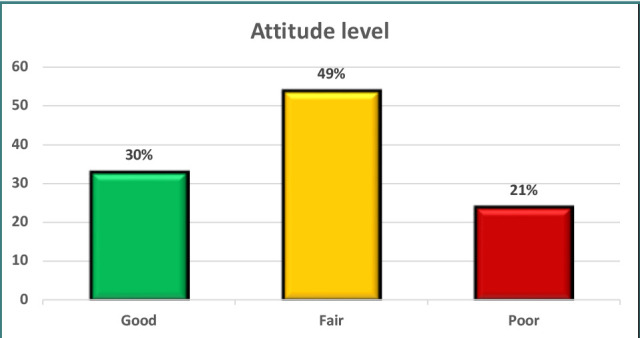
Distribution of attitude levels across participants

A significant proportion of patients (30%) had a positive attitude, whereas 49% showed a neutral attitude, and 21% revealed a negative attitude (Figure 2).

Approximately 94.6% of patients consistently adhered to their drug regimen (Table 4). All participants in the study consistently adhered to the prescribed regimen of consuming levothyroxine around 30–60 minutes before breakfast without concomitant administration of any other medications. Most patients (58.6%) lacked knowledge regarding the recommended avoidance of cabbage, cauliflower, and soy in their diet.

**Table 4:** Patient’s practice responses

	Yes		No	
	*n*	%	*n*	%
Do you regularly take your hypothyroidism medication?	105	94.6	6	5.4
Do you ever forget to take your hypothyroidism medication?	54	48.6	57	51.4
Do you take your medication 30–60 minutes before breakfast on an empty stomach?	111	100.0	0	0.0
Do you use any other medications along with your thyroid medication?	0	0.0	111	100.0
Do you regularly check your TSH level as your doctor has instructed?	73	65.8	38	34.2
Do you look for information on hypothyroidism on the internet or your smartphone?	65	58.6	46	41.4
Did you ask your doctor for more information or counseling on how to manage hypothyroidism?	59	53.2	52	46.8
Do you avoid eating cabbage, cauliflower, and soy?	46	41.4	65	58.6

Data presented as number (*n*) and percentage (%)

**Figure 3:**
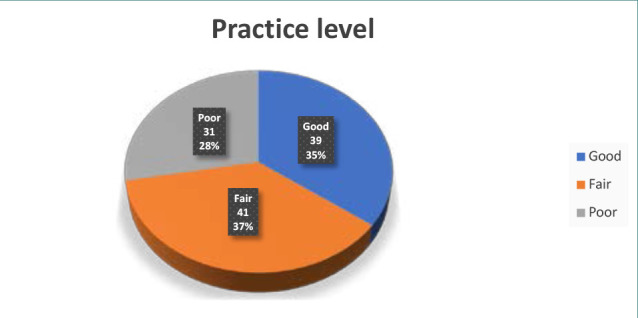
Distribution of participants according to practice level

Figure 3 reveals that 35% of the patients had good practice managing their condition. In addition, 41% demonstrated fair practice, and 28% showed poor practice in managing their condition.

**Figure 4:**
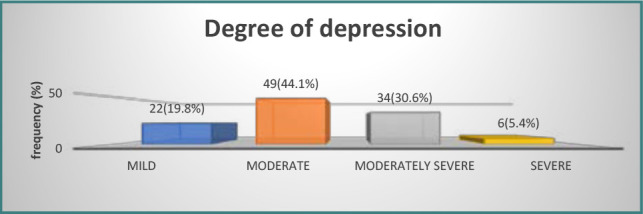
Distribution of patients according to depression level

Table 5 categorizes participant responses to depression-related questions. Mild depression was present in 19.8% of patients, 44.1% experienced moderate depression, 30.6% moderately severe depression, and 5.4% demonstrated severe depression (Figure 4).

**Table 5:** Patient’s depression responses

Questions	Not at all		Several days		More than half the day		Nearly every day	
	*n*	%	*n*	%	*n*	%	*n*	%
Little interest or pleasure in doing things	100	90.1	6	5.4	1	0.9	4	3.6
Feeling down, depressed, or hopeless	3	2.7	28	25.2	66	59.5	14	12.6
Trouble falling or staying asleep or sleeping too much	5	4.5	22	19.8	66	59.5	18	16.2
Feeling tired or having little energy	14	12.6	35	31.5	44	39.6	18	16.2
Poor appetite or overeating	5	4.5	15	13.5	62	55.9	29	26.1
Feeling bad about yourself — or that you are a failure or have let yourself or your family down	64	57.7	30	27.0	14	12.6	3	2.7
Trouble concentrating on things, such as reading the newspaper or watching television	59	53.2	36	32.4	11	9.9	5	4.5
Moving or speaking so slowly that other people could have noticed? Or the opposite — being so fidgety or restless that you have been moving around a lot more than usual	2	1.8	8	7.2	42	37.8	59	53.2
Thoughts that you would be better off dead or hurting yourself in some way	14	12.6	21	18.9	52	46.8	24	21.6

Data presented as number *(n*) and percentage (%)

**Figure 5:**
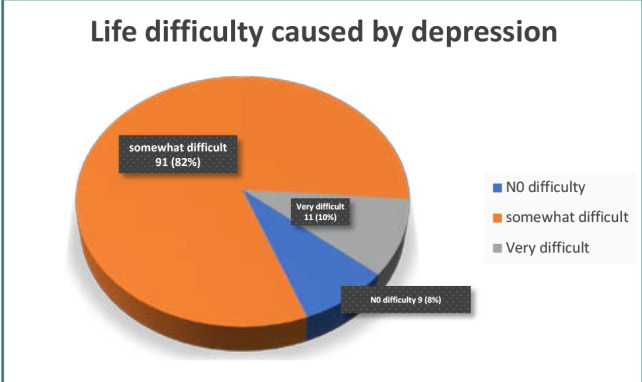
distribution of patients according to life difficulty due to depression

The data shown in Figure 5 indicates that few patients (8%) experienced no difficulties in their lives due to depression. The majority (82%) encountered some level of difficulty in their daily lives. Additionally, 10% of patients reported facing major difficulties due to depression.

**Figure 6:**
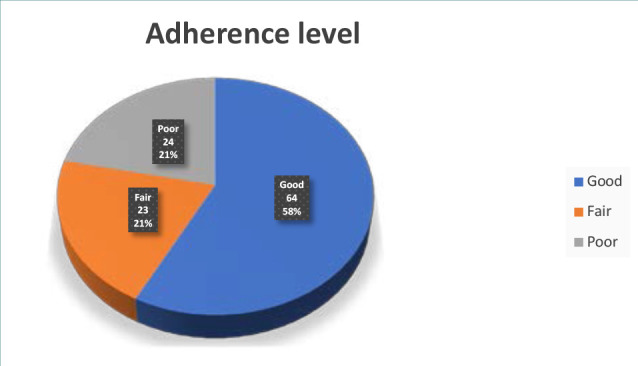
Distribution of participants according to adherence level

The majority of patients (58%) had excellent adherence, while a smaller proportion had fair adherence (21%) and poor adherence (21%) (Figure 6).

### Association between patient demographics and subjective assessment

The chi-square test indicated no statistically significant relationship between the degree of knowledge, attitudes, and practices (KAP) and age or gender (Table 6) (*P* > 0.05). Participants with a greater level of education had a more favorable level of knowledge, attitudes, and practices than those with a lower educational level (*P* < 0.05). There was no statistically significant correlation seen between the severity of depression, life challenges, age, or level of education across all conditions (*P* > 0.05). Female patients had a significantly higher level of depression and a greater incidence of difficulties (*P* values of 0.002 and 0.020, respectively).

**Table 6:** Association between patient demographics and subjective assessment

			Age (years)				Gender			Education			
			<40 *n* (%)	40-60 *n* (%)	>60 *n* (%)	*P* value	Male *n* (%)	Female *n* (%)	*P* value	Primary n (%)	Secondary *n* (%)	University *n* (%)	*P* value
**Knowledge level**		**Good *n* = 28**	12 (28.6)	14 (25.5)	2 (14.3)	0.415 NS	3 (30.0)	25 (24.8)	0.776 NS	5 (17.2)	9 (17.6)	14 (45.2)	0.043^*^
		**Fair *n* = 45**	17 (40.5)	24 (43.6)	4 (28.6)		3 (30.0)	42 (41.6)		12 (41.4)	22 (43.1)	11 (35.5)	
		**Poor *n* = 38**	13 (31.0)	17 (30.9)	8 (57.1)		4 (40.0)	34 (33.7)		12 (41.4)	20 (39.2)	6 (19.4)	
**Attitude level**		**Good *n* = 33**	14 (33.3)	15 (27.3)	4 (28.6)	0.775 NS	4 (40.0)	29 (28.7)	0.138 NS	8 (27.6)	11 (21.6)	14 (45.2)	0.032^*^
		**Fair *n* = 54**	18 (42.9)	30 (54.5)	6 (42.9)		2 (20.0)	52 (51.5)		13 (44.8)	25 (49.0)	16 (51.6)	
		**Poor *n* = 24**	10 (23.8)	10 (18.2)	4 (28.6)		4 (40.0)	20 (19.8)		8 (27.6)	15 (29.4)	1 (3.2)	
**Practice level**		**Good *n* = 39**	16 (38.1)	19 (34.5)	4 (28.6)	0.073 NS	6 (60.0)	33 (32.7)	0.128 NS	8 (27.6)	14 (27.5)	17 (54.8)	0.005^**^
		**Fair *n* = 41**	9 (21.4)	25 (45.5)	7 (50.0)		1 (10.0)	40 (39.6)		11 (37.9)	17 (33.3)	13 (41.9)	
		**Poor *n* = 31**	17 (40.5)	11 (20.0)	3 (21.4)		3 (30.0)	28 (27.7)		10 (34.5)	20 (39.2)	1 (3.2)	
**Adherence level**		**Good *n* = 64**	22 (52.4)	34 (61.8)	8 (57.1)	0.585 NS	4 (40.0)	60 (59.4)	0.495 NS	16 (55.2)	26 (51.0)	22 (71.0)	0.360 NS
		**Fair *n* = 23**	11 (26.2)	8 (14.5)	4 (28.6)		3 (30.0)	20 (19.8)		6 (20.7)	11 (21.6)	6 (19.4)	
		**Poor *n* = 24**	9 (21.4)	13 (23.6)	2 (14.3)		3 (30.0)	21 (20.8)		7 (24.1)	14 (27.5)	3 (9.7)	
**Depression**	**Degree**	**Mild**	13 (31.0)	6 (10.9)	3 (21.4)	0.166 NS	6 (60.0)	16 (15.8)	0.002^**^	4 (13.8)	7 (13.7)	11 (35.5)	0.141 NS
		**Moderate**	15 (35.7)	27 (49.1)	7 (50.0)		4 (40.0)	45 (44.6)		13 (44.8)	24 (47.1)	12 (38.7)	
		**Severe**	14 (33.3)	22 (40.0)	4 (28.6)		0 (0.0)	40 (39.6)		12 (41.4)	20 (39.2)	8 (25.8)	
	**Life** **difficulty**	**No difficulty**	6 (14.3)	2 (3.6)	1 (7.1)	0.164NS	3 (30.0)	6 (5.9)	0.020^*^	3 (10.3)	1 (2.0)	5 (16.1)	0.167 NS
		**Some difficulty**	33 (78.6)	45 (81.8)	13 (92.9)		7 (70.0)	84 (83.2)		22 (75.9)	46 (90.2)	23 (74.2)	
		**Very difficult**	3 (7.1)	8 (14.5)	0 (0.0)		0 (0.0)	11 (10.9)		4 (13.8)	4 (7.8)	3 (9.7)	

Data presented as numbers (n) and percentages (%). Chi-square test comparing categorical variables; P value ≥ 0.05, NS, non-significant. *(P < 0.05), significant, **(P < 0.01), highly significant

There was a statistically significant relationship between TSH levels and the levels of knowledge, practice, attitude, and adherence (Table 7).

**Table 7:** Correlation between TSH level and subjective assessment

		Knowledge level	Practice level	Adherence level	Attitude level	Depression. score
TSH mIU/L	R	-0.230-^*^	-0.299-^**^	-0.372-^**^	-0.266-^**^	-0.090-
	Pv	0.015^*^	<0.01**	<0.01**	0.005**	0.348
Knowledge level	R		0.377^**^	0.203^*^	0.563^**^	-0.025-
	Pv		<0.01**	0.032	<0.01**	0.793
Practice level	R			0.571^**^	0.458^**^	0.096
	Pv			<0.01**	<0.01**	0.317
Adherence level	R				0.310^**^	0.145
	Pv				0.001**	0.128
Attitude level	R					0.005
	Pv					0.956
Depression. Score	R					
	Pv					

Correlation coefficient (r), ** Correlation was highly significant at the P value < 0.01 level, milli-international units per liter (mIU/L)

Approximately 25% of participants had a high degree of knowledge, although a greater number (41%) showed moderate knowledge. The remaining participants (34%) had a poor level of knowledge. In what concerns attitudes, 30% of participants had a positive attitude, 49% had a neutral attitude, and 21% showed an unfavorable attitude. In addition, 35% demonstrated adherence to recommended practices. Furthermore, a notable proportion of patients (37%) had fair practice, while 28% had poor practice. The results also revealed that 58% of the patients adhered well to their prescribed treatment regimens, whereas only 21% showed acceptable adherence, and another 21% displayed poor adherence (Table 8).

**Table 8:** Distribution of participants according to KAP and adherence levels

KAP and adherence	Good		Fair		Poor	
	*n*	%	%	*n*	%	*n*
**Knowledge level**	28	25	45	41	38	34
**Attitude level**	33	30	54	49	24	21
**Practice level**	39	35	41	37	31	28
**Adherence level**	64	58	23	21	24	21

Data presented as number (n) and percentage (%)

## Discussion

The majority of participants (91.0%) in this study were women. Moreover, the prevalence of the condition increased with age, peaking among individuals aged 40 to 60. This finding aligns with a study from India involving 312 participants with thyroid dysfunction, with an increased prevalence in women (87.49%) compared to men (12.49%). This study also found hypothyroidism to be more common in women aged 30 to 45 years, further emphasizing the gender and age-related variations in thyroid dysfunction rates within the Indian population [22,23]. Similarly, in a recent cross-sectional study conducted in Iraqi Kurdistan, primary hypothyroidism was significantly more prevalent in women compared to men, with a relative risk of 8-9 [24,25].

Regarding obesity, 36% of patients in our research were classified as obese, aligning with the data obtained from a previous study conducted in Basrah, which also reported a high prevalence of obesity among patients with hypothyroidism [26]. Most patients in our study had a secondary level of education, which contrasts with findings from Saudi Arabia [27] and India [4]. In Saudi Arabia, 61% of the participants had an undergraduate level of education [27], while in India, over 53.6% had a bachelor or postgraduate degree [4]. Additionally, in another study, 55.8% of the individuals held graduate or postgraduate degrees in other areas of study [15]. In our study, most participants (65.8%) had a family history of thyroid disorders. This is comparable with another study where 63% of respondents had a family history of thyroid diseases [28].

A significant proportion of patients had hypertension and diabetes in our study, supporting other studies that highlight a correlation between hypothyroidism and many health conditions such as asthma, obesity, diabetes, and hypertension [29,30]. Both hypothyroidism and hyperthyroidism increase the likelihood of developing hypertension [31]. Several studies have shown a positive correlation between subclinical hypothyroidism and hypertension in women [32,33]. Data from the National Health and Nutrition Examination Survey (NHANES III) in the United States revealed that individuals with diabetes are more prone to thyroid dysfunction, especially those with thyroid peroxidase antibodies (TPOAb) [34].

In this study, only 25% of patients had a high level of knowledge, while the majority had a moderate (41%) to poor level of knowledge (34%). This contrasts with a study from Saudi Arabia in 2023, where only 9% of participants had a high level of knowledge, while the remaining 91% had a low level of understanding [35]. Other research in Saudi Arabia indicated that half of the respondents had a moderate knowledge level [36], but only 14.2% showed a satisfactory understanding of the condition [37]. Thyroid illness is a prevalent condition that often presents without noticeable symptoms. However, if left untreated, the condition may give rise to significant complications [38]. The majority of patients involved in the study had a high level of knowledge regarding the thyroid gland anatomy, as well as hypothyroidism. Previous studies have reported similar findings regarding patients' knowledge. For instance, a study conducted in India revealed that over 85% of individuals with hypothyroidism were aware of the diagnostic significance of thyroid-stimulating hormone. Moreover, a significant majority (91.4%) recognized the necessity of consulting a physician before beginning treatment [15]. However, a study at Teerthanker Mahaveer Hospital revealed that 14% were unaware of the anatomical positioning of the thyroid gland [30].

Furthermore, it is worth noting that only 14% of the participants in the current research were aware of the contraindications associated with the concurrent use of thyroid medications and iron and calcium supplements. This is supported by Mazokopakis *et al*., who found that only 8.4% of 153 individuals took calcium carbonate at least 4 hours apart from LT4 [39]. Furthermore, a considerable number of participants lacked an understanding of the association between hypothyroidism and elevated cholesterol levels. Enhancing patient knowledge about the potential reduction in total cholesterol levels through effective hypothyroid treatment could increase the value of levothyroxine therapy [15,40]. Furthermore, the patients included in the present research had a favorable attitude ranging from excellent to fair, with a prevalence of 30% to 49%. Both groups had similar levels of practice, with 35% and 37%, respectively, categorized as good to fair, confirming previous findings [4].

The study also highlighted a widespread belief among participants that women, being more susceptible to hypothyroidism, should undergo regular thyroid function tests—a view supported by 71% of respondents in recent research, while only 24.90% disagreed [23]. It is important to note that the self-adjustment of medication dosages is not advisable in the context of hypothyroidism. About 41.4% of patients showed proficient adherence, understanding the potential effects of foods like cabbage, cauliflower, and soy. This aligns with other studies that indicated a considerable awareness among patients about the effects of goitrogenic foods on thyroid function [28,41,42]. In another study, a comparable proportion of individuals (91.5%) from Saudi Arabia consistently followed their prescribed treatment regimen daily [27].

Several factors contribute to the gap in patient understanding of thyroid disorders, including a scarcity of adequately trained physicians, less time doctors give for patient education because of heavy patient burden, insufficient patient knowledge, and underutilization of electronic media tools [43]. This underscores the urgent need for specialized endocrinology healthcare professionals, including endocrine nurses, counselors, and dietitians, to enhance patient understanding and management of thyroid disorders [4].

A significant proportion of participants in our study had symptoms of depression ranging from mild to quite severe, supporting the findings of Mohammad *et al*., where 33.9% of patients received a diagnosis of depression with different degrees of severity [44]. Similar rates were reported in studies conducted in Nepal and Turkey, indicating that 24% of individuals diagnosed with thyroid disorders also had symptoms of depression [45]. Individuals diagnosed with severe schizophrenia (SCH) have a higher susceptibility to engaging in suicidal behaviors and manifesting mental symptoms [46]. Imbalances in thyroid hormone levels, whether insufficient or excessive, may lead to a wide array of neuropsychiatric symptoms, including depression [47]. This study found that 82% of participants faced significant challenges due to their depressive condition. Highlighting the impact of thyroid health on quality of life, research indicated that higher TSH levels were associated with lower health-related quality of life (HRQL), suggesting a direct correlation between TSH levels and experiences of fatigue and emotional sensitivity [48].

This study observed good adherence to levothyroxine therapy, contrasting with previous research reporting a 66.7% low adherence rate [49]. Our findings also revealed a positive correlation between education level and knowledge about hypothyroidism (*P* = 0.043). Patients with university or secondary education demonstrated better knowledge than those with primary education. Interestingly, some studies suggest that higher educational attainment might lead to increased worry and a tendency to take more safety precautions [15]. This suggests that increasing awareness and understanding of thyroid disease among those diagnosed can significantly improve treatment adherence and reduce morbidity [50]. A study suggested that enhanced knowledge and understanding of thyroid disease encouraged patients to adhere to their prescribed medication regimen [30].

Female participants exhibit a twofold higher susceptibility to the onset of serious depression compared to male participants [51]. The variations in prevalence can be attributed to dissimilarities in sample size, gender distribution, and additional socioeconomic variables among the several populations under investigation [44]. Management of hypothyroidism typically involves a consistent daily dose of levothyroxine, which effectively normalizes serum TSH levels [7]. Interestingly, this study found no significant correlation between TSH levels and depression symptoms, aligning with previous research [45,52,53]. However, Panicker *et al*. [45] reported a significant interaction between TSH and depression. The differences observed between the findings of the current research and other studies might be attributed to factors like the small sample size and the specific conditions under which the research was conducted. Subsequently, longitudinal studies are needed to explain the disparities in the literature.

## LIMITATIONS

The present study has certain limitations that should be acknowledged. The study recruited participants from a single medical facility in Iraq, limiting the generalizability of findings to the broader Iraqi population. Consequently, A relatively small cohort was evaluated for KAP (Knowledge, Attitudes, Practices), treatment adherence, and depression. Furthermore, relying on researcher-reported responses instead of self-reported data raises potential concerns about bias, such as inaccurate recall or social desirability bias.

## CONCLUSION

The study highlights that despite high adherence to the prescribed treatment, factors such as knowledge, attitudes, practices, and moderate to severe depression negatively influenced the efficacy of replacement therapy. The prevalence of primary hypothyroidism was higher among women who were overweight, experienced substantial depressive symptoms, and faced challenging life circumstances as compared to males. Patients with a higher education level demonstrated superior levels of awareness and adherence to recommended practices compared to those with a lower educational level.

## Personal thanks

The authors gratefully acknowledge Mustansiriyah University, Baghdad, Iraq, for granting approval to conduct this study. We also extend our sincere appreciation to all participants for their valuable contributions.
